# Critical Limb Ischemia Induces Remodeling of Skeletal Muscle Motor Unit, Myonuclear-, and Mitochondrial-Domains

**DOI:** 10.1038/s41598-019-45923-4

**Published:** 2019-07-02

**Authors:** Mahir Mohiuddin, Nan Hee Lee, June Young Moon, Woojin M. Han, Shannon E. Anderson, Jeongmoon J. Choi, Eunjung Shin, Shadi A. Nakhai, Thu Tran, Berna Aliya, Do Young Kim, Aimee Gerold, Laura M. Hansen, W. Robert Taylor, Young C. Jang

**Affiliations:** 1 School of Biological Sciences, Georgia Institute of Technology, Atlanta, GA 30332 USA; 2 Wallace H. Coulter Department of Biomedical Engineering, Georgia Institute of Technology and Emory University, Atlanta, GA 30332 USA; 3 Parker H. Petit Institute for Bioengineering and Bioscience, Georgia Institute of Technology, Atlanta, GA 30332 USA; 4 George W. Woodruff School of Mechanical Engineering, Georgia Institute of Technology, Atlanta, GA 30332 USA; 5 Division of Cardiology, Emory University School of Medicine, Atlanta, GA 30322 USA

**Keywords:** Mechanisms of disease, Peripheral vascular disease, Muscle stem cells, Muscle stem cells, Stem-cell niche

## Abstract

Critical limb ischemia, the most severe form of peripheral artery disease, leads to extensive damage and alterations to skeletal muscle homeostasis. Although recent research has investigated the tissue-specific responses to ischemia, the role of the muscle stem cell in the regeneration of its niche components within skeletal muscle has been limited. To elucidate the regenerative mechanism of the muscle stem cell in response to ischemic insults, we explored cellular interactions between the vasculature, neural network, and muscle fiber within the muscle stem cell niche. Using a surgical murine hindlimb ischemia model, we first discovered a significant increase in subsynaptic nuclei and remodeling of the neuromuscular junction following ischemia-induced denervation. In addition, ischemic injury causes significant alterations to the myofiber through a muscle stem cell-mediated accumulation of total myonuclei and a concomitant decrease in myonuclear domain size, possibly to enhance the transcriptional and translation output and restore muscle mass. Results also revealed an accumulation of total mitochondrial content per myonucleus in ischemic myofibers to compensate for impaired mitochondrial function and high turnover rate. Taken together, the findings from this study suggest that the muscle stem cell plays a role in motor neuron reinnervation, myonuclear accretion, and mitochondrial biogenesis for skeletal muscle regeneration following ischemic injury.

## Introduction

Peripheral artery disease (PAD) is an age-associated degenerative vascular disease characterized by abnormal perfusion in the limbs due to occlusions of the blood vessels. In its most severe form, referred to as critical limb ischemia (CLI), where blood flow is insufficient to maintain tissue viability, amputation rates can reach up to 50%^1^. While recent regenerative medicine approaches on collateral vessel formation have made some progress, the dysregulation of the skeletal muscle in CLI and the subsequent tissue remodeling have not been thoroughly investigated^2–4^. Recent evidence suggests that the ischemia-induced loss of viable muscle tissue, which acts as an indispensable matrix of growth factors and biomechanical support for new vessel formation, may be the cause of failure for current revascularization therapies^5,6^. This paradox emphasizes the importance to elucidate the skeletal muscle regeneration mechanism in complex injuries such as CLI.

Skeletal muscles possess a remarkable regenerative capacity due to the presence of endogenous resident muscle stem cells (MuSCs), also known as satellite cells^7^. Upon injury, Pax7^+^ quiescent MuSCs, which reside adjacent to basal lamina and sarcolemma of the multinucleated myofiber, undergo asymmetric division in which some committed progenies can either form *de novo* myofibers to generate additional myofibers or proliferate, differentiate, and fuse with existing myofibers to increase the number of myonuclei and volume in regenerating fibers. Meanwhile, other populations of MuSC progeny self-renew to replenish the quiescent stem cell pool for future rounds of regeneration^8^. Emerging evidence suggests that MuSC function and regenerative capacity are dictated by cellular and acellular interactions with its surrounding microenvironment, or niche, such as the vasculature, neuromuscular junction (NMJ) of a neural network, myofibers, interstitial stromal cells of the extracellular matrix, as well as various infiltrating immune cells^9^. Of these niche components, the vasculature and microvessels are located close to MuSCs and provide necessary nutrients and growth factors required for MuSC function and muscle homeostasis^10^. Blood vessels also carry oxygen and carbon dioxide that are essential for oxidative metabolism throughout the myofibers and within MuSCs to generate energy and regulate redox signaling^11,12^. In addition, vascular networks near MuSCs play a crucial role in recruiting circulating stem cells and transporting immune cells during the initial phase of muscle repair^13,14^. Thus, the lack of functional blood perfusion to skeletal muscle not only disrupts cellular function and respiration by limiting nutrient and oxygen delivery but also compromises muscle regeneration^15^.

It has been well documented that motor neuron innervation maintains muscle homeostasis by regulating excitation-contraction coupling as well as controlling the gene expression pattern of myofibers^16^. Denervation of the muscle fiber due to injury or neuromuscular disease results in muscle wasting and remodeling of motor units^17,18^. Anatomically, peripheral nerves, such as lower motor neurons, are located close in contact with the vasculature (neurovascular congruency)^19^ and exhibit functional interdependency. In support of this notion, some of the axonal guidance factors are known to possess angiogenic properties^20^, and other well-known angiogenic factors, such as vascular endothelial growth factor (VEGF), guide terminal Schwann cell-mediated peripheral nerve regeneration^21^. As such, when blood flow is restricted in ischemic injury, motor neurons undergo rapid Wallerian degeneration, and their regenerative response is activated^22,23^. Moreover, a recent report showed that the regeneration of the motor neuron and its corresponding neuromuscular synapses are in part linked to an increase in activation of MuSC and myogenesis, supported by the proximity of the MuSC to the NMJ^24^. Conversely, genetic depletion of MuSCs diminishes the regenerative response of the NMJ^25^, demonstrating crosstalk between the MuSC and the NMJ. Furthermore, it has been revealed that synaptic mitochondria also play a role in maintaining NMJ^26,27^. While ischemic injury has been linked to excessive generation of the deleterious mitochondrial-derived reactive oxygen species (ROS) that oxidize the NMJ and other niche components of the MuSC, protecting mitochondria against oxidative stress attenuates ischemia-induced denervation and muscle atrophy^23^. Yet, the majority of previous studies have reported tissue-specific interactions in response to ischemic insults (i.e., ischemia – muscle stem cell, ischemia – muscle fiber, ischemia – motor neuron), and a comprehensive investigation elucidating the mechanistic crosstalk between collateral vascularization, motor unit formation, muscle stem cell activation, muscle fiber regeneration, and the mitochondria that drive these energy-demanding processes altogether has not been conducted.

To address this gap in knowledge, we characterized the remodeling of the MuSC niche components, notably the vasculature, NMJ, and myofiber, at various time points to elucidate the sequential regenerative response following ischemia/reperfusion injury. Using the murine hindlimb ischemia (HLI) model that manifests similar pathophysiology as human CLI, here we demonstrate ischemia-induced early necrotic degeneration of muscle fibers and denervation of the NMJ. First, we show that an increase in subsynaptic nuclei and myonuclei throughout the muscle fiber are driven by proliferation and activation of MuSCs, which facilitate the regeneration of these tissues. Second, the accretion of myonuclei produces a smaller myonuclear domain, or cytoplasmic volume of myofiber transcriptionally governed by each myonucleus, as a regenerative response to ischemia. In parallel, the increased myogenesis and myonuclear number are coupled with an increase in mitochondrial content that is compartmentalized into discrete domains around each myonucleus. Moreover, we show that the high turnover rate of mitochondria through autophagy at early stages of regeneration resets the mitochondrial domain, or the mitochondrial network surrounding each myonucleus. Collectively, this study highlights the highly orchestrated remodeling of the MuSC and its niche components following CLI and provides a basis to investigate multi-scale therapies for complex skeletal muscle injuries.

## Results

### Hindlimb ischemia induces skeletal muscle regeneration

**Figure 1:**
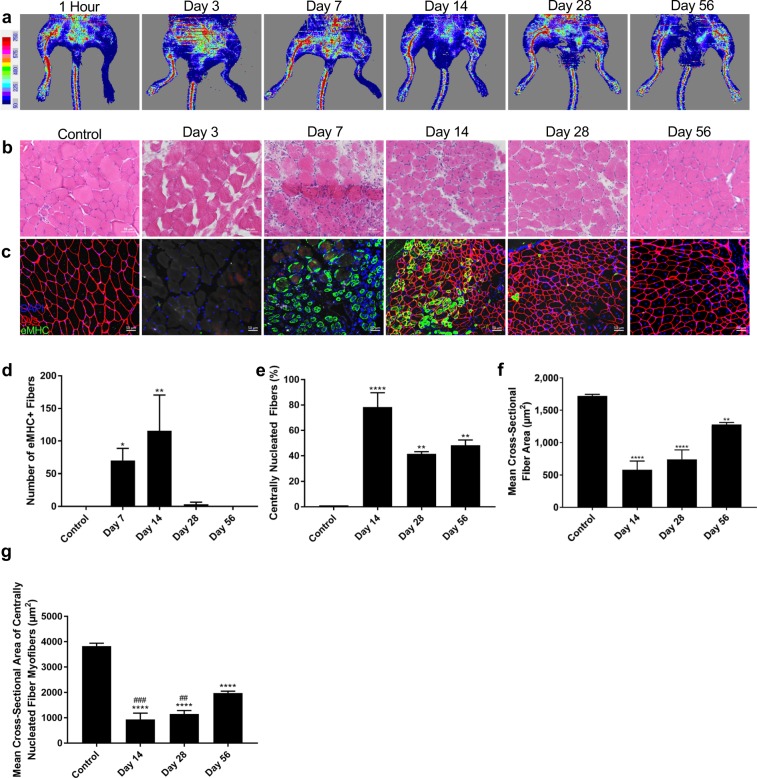
Characterization of CLI mouse model and skeletal muscle regeneration. (**a**) LDPI of ventral mouse hindlimbs 1 hour, 3 days, 7 days, 14 days, 28 days, and 56 days following CLI. Control leg on left, ischemic leg on right. Scale bar represents blood flow perfusion by color. (**b**) H&E staining of TA cross-sections in control, and 3 days, 7 days, 14 days, 28 days, and 56 days following CLI. (**c**) Immunohistochemistry of TA cross-sections in control, and 3 days, 7 days, 14 days, 28 days, and 56 days following CLI. Dystrophin pseudo-colored in red, eMHC in green, nuclei in blue. Scale bars on cross-sections represent 50 µm. (**d**) Total number of eMHC+ fibers within a 0.33 mm^2^ field of view for control, 7 days, 14 days, and 56 days following CLI. (**e**) Percentage of centrally nucleated fibers in control, 14 days, 28 days, and 56 days following surgery. (**f**) Mean cross-sectional fiber area of 4 random 0.33 mm^2^ fields of view of the TA using dystrophin in control, 14 days, 28 days, and 56 days following CLI. n = 3, ^*^p < 0.05, **p < 0.01, ***p < 0.001, ****p < 0.0001 compared to control for all figures. (**g**) Mean cross-sectional area of centrally nucleated myofibers of the TA. n = 3, ****p < 0.0001 compared to control. ^##^*p* < 0.01, ^###^*p* < 0.001 compared to day 56.

To investigate morphological changes to the skeletal muscle fibers and muscle stem cell niche following chronic hindlimb ischemia (HLI), we first correlated blood perfusion to muscle regeneration. Laser Doppler perfusion imaging (LDPI) was used to quantify the abnormal perfusion in the ischemic limb (right-hand side of each scan) to demonstrate a significant decrease in perfusion to the ischemic limb compared to its contralateral control (left-hand side of each scan) at all time points up to 56 days following HLI (Figs 1a and S1a). As expected, hematoxylin-eosin (Fig. 1b) and immunofluorescent staining (Fig. 1c) of cross-sections from the tibialis anterior muscle (TA) showed extensive myofiber damage, and presence of necrotic fibers markedly increased at day 3 and 7. A high number of nuclei within the interstitial space was also observed at 7 days following HLI, implying immune cell infiltration required for clearance of dead or damaged myofibers^14^. Reperfusion to the ischemic limb did not improve until day 14 (Supplementary Fig. S1b), where we found a coinciding peak in embryonic myosin heavy chain (eMHC) positive myofibers (Fig. 1d), a marker for early stages of regeneration^28^. This suggests that sufficient perfusion through collateral vascularization parallels activation of muscle stem cells and muscle regeneration. To further assess myofiber regeneration, we noted a substantial increase in the percentage of centrally nucleated fibers, characteristic of regenerating myofibers, at day 14 that persisted for up to 56 days (Fig. 1e). Furthermore, even though muscle fibers are regenerating, muscle atrophy, as measured by overall fiber cross-sectional area, did not fully recover until day 56, suggesting that the functional deficiency may be prolonged following ischemic myopathy (Fig. 1f). By measuring the cross-sectional area of only centrally nucleated myofibers, we report a decrease in area of regenerating muscle fibers at days 14 and 28 followed by a subsequent increase in the area by day 56 that is still not fully healed compared to control (Fig. 1g). In accordance with other models of ischemia^15^, we observed a delayed regenerative response to ischemia compared to chemical modes of injury such as cardiotoxin and barium chloride, where immune cell infiltration was seen at days 3–4 and both eMHC^+^ and centrally nucleated fibers were decreased by day 14 following injury^29,30^. These data show a delayed ischemia-induced regenerative response of skeletal muscle tissue, dependent on reperfusion to the hindlimb, which continues for at least 56 days.

### Hindlimb ischemia induces motor unit remodeling

**Figure 2:**
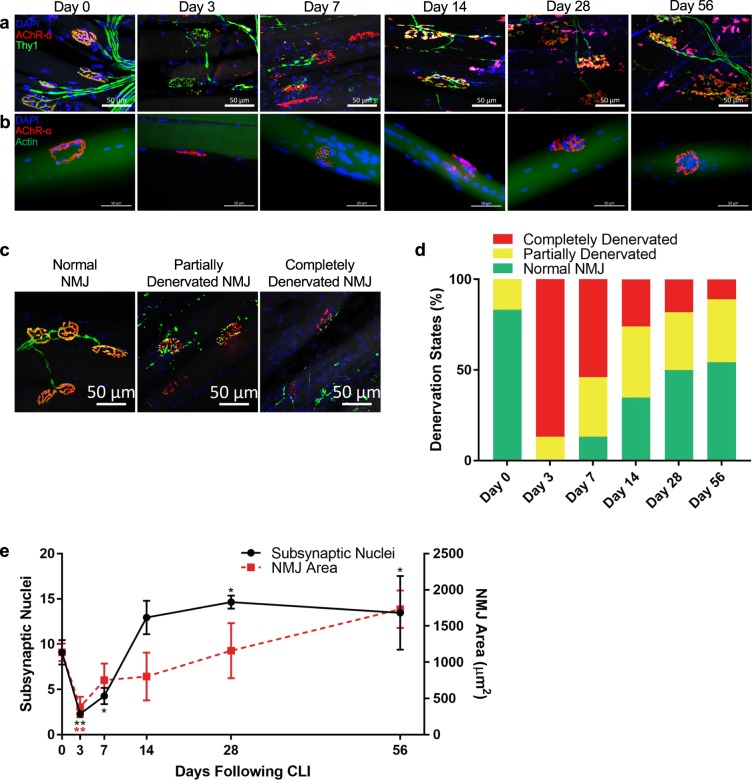
Remodeling of the motor unit. (**a**) NMJ in EDL 1 hour (day 0), 3 days, 7 days, 14 days, 28 days, and 56 days following CLI. Nuclei pseudo-colored in blue, α-bungarotoxin (for acetylcholine receptor-α subunit) in red, Thy1 (for motor neuron axon terminal) in green. Maximum intensity projection was performed on images from confocal microscopy. (**b**) NMJ on single myofibers from TA from 1 hour (day 0), 3 days, 7 days, 14 days, 28 days, and 56 days following CLI. Nuclei pseudo-colored in blue, acetylcholine receptor-α subunit in red, actin in green. (**c**) Representative images of normal, partially denervated, and completely denervated NMJ. Acetylcholine receptor-α subunit (α-bungarotoxin) pseudo-colored in red, Thy1 in green. (**d**) Percentages of normal, partially denervated, and completely denervated NMJ in EDL (at least 20 NMJs per sample) 1 hour (day 0), 3 days, 7 days, 14 days, 28 days, and 56 days following CLI. (**e**) Number of subsynaptic myonuclei within each NMJ (at least 15 NMJ’s per sample) and their associated NMJ areas of single myofibers from the TA. All scale bars represent 50 µm. n = 3, *p < 0.05, **p < 0.01, ***p < 0.001, ****p < 0.0001 compared to day 0 for all figures.

In the next set of experiments, we examined the effects of ischemia on motor neuron disruption and subsequent denervation at the neuromuscular junction (NMJ). Given that a decline in innervation and loss of motor units are known causes of muscle degeneration^31^, we tested whether denervation plays a role in the ischemia-induced atrophic process. Using a well-established motor neuron reporter mice, in which YFP expression is driven by the *Thy1* regulatory element^32^, we observed marked alterations in NMJs in the ischemic extensor digitorum longus muscle (EDL) compared to control (Fig. 2a). Notably, at day 3 and day 7, presynaptic terminals of the motor axons exhibited abnormal thinning and characteristics of Wallerian degeneration while the postsynaptic endplates were severely fragmented compared to the normal pretzel-like morphology seen at day 0. When muscle fibers are innervated, the presynaptic nerve terminal and postsynaptic acetylcholine receptors (AChR) of the NMJ overlay each other. Hence, to quantify innervation states of the muscle fibers, NMJs of each group were categorized as normal NMJ morphology (pretzel-like, convoluted folding structure with more than 75% overlay), partially denervated (fragmented AChR with between 25% and 75% overlay), or completely denervated (fragmented AChR with less than 25% overlay). Representative images of denervation states are shown in Fig. 2c. Following HLI, we report a decrease in NMJs with normal morphology and an increase in complete denervation in ischemia-affected muscle compared to day 0, with peak denervation observed on day 3. Interestingly, the loss of normal NMJ morphology lasted until at least day 56 (Figs 2d and S2a). These data further suggest a persistent functional deficiency in myofiber excitation-contraction coupling and corroborates the continued regeneration of skeletal muscle tissues up to 56 days.

Next, we performed analyses on single myofibers randomly isolated from TA muscle of control and HLI, comprised of almost all fast-twitch oxidative-glycolytic (type IIA) and fast-twitch glycolytic (types IIB/X) with few slow-twitch oxidative (type I) fibers^33^, to reveal any remodeling of the NMJ endplates at the individual muscle fiber level. It is worth noting that since fiber type is in part determined by innervation from a specific motor neuron type, alteration in the motor unit organization may lead to a shift in fiber type^34^. Indeed, we observed an ischemia-induced loss of slow-twitch type I fibers 14- and 28-days following injury while percentages of fast-twitch type IIA and types IIB/X fibers were unchanged (Supplementary Fig. S2b–d). Regardless of the shift in fiber type and due to the pervasive fragmentation of AChRs observed in isolated myofibers (Fig. 2b), we counted the subsynaptic nuclei that are responsible for maintenance of the NMJ through regulation of gene expression^35^. Despite a transient decrease in subsynaptic nuclei per NMJ at days 3 and 7, which may be attributed to smaller, fragmented NMJs in denervated myofibers^36^, we observed a significant increase in the number of subsynaptic nuclei 28 and 56 days following HLI compared to day 0 (Fig. 2e). Intriguingly, the accumulation of subsynaptic nuclei precedes the increase in NMJ area. The increased subsynaptic nuclei also coincide with the regeneration of the NMJ, indicating a dependence of NMJ restoration on the number of subsynaptic nuclei. Therefore, results suggest that ischemia-induced denervation plays a role in muscle atrophy and the remodeling of subsynaptic nuclei assist in the repair of denervated NMJ for up to 56 days following HLI.

### Muscle stem cell decreases myonuclear domain following hindlimb ischemia

**Figure 3:**
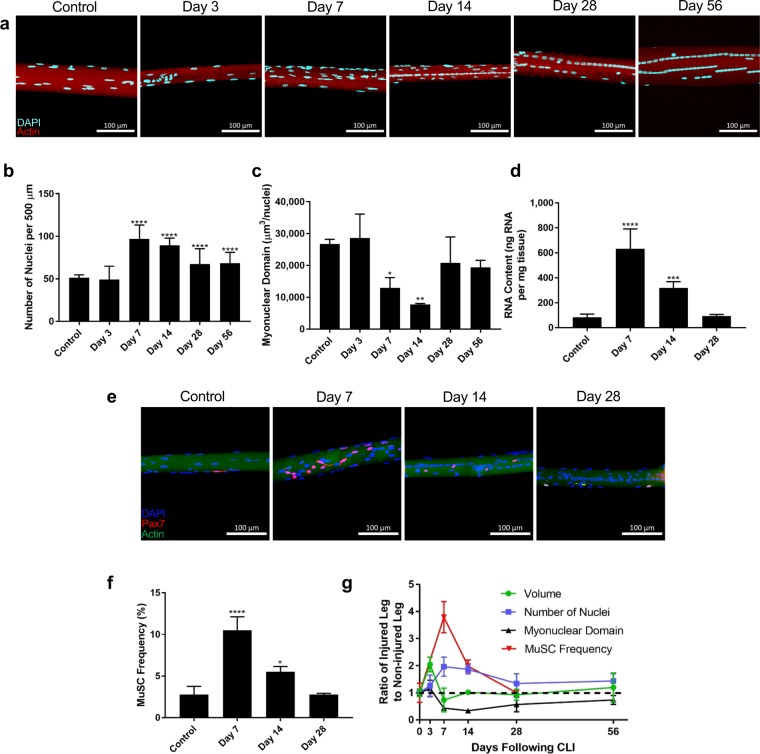
Changes in myonuclear domain following CLI. (**a**) Z-stack confocal imaging of single myofibers from TA of control, 3 days, 7 days, 14 days, 28 days, and 56 days following CLI. Nuclei pseudo-colored in light blue, actin in red. (**b**) Number of myonuclei per 500 µm of myofiber (at least 20 fibers per sample) at various timepoints (n = 6). (**c**) Myonuclear domain of at least 20 single myofibers per sample over 500 µm at various timepoints, calculated as myofiber volume divided by number of myonuclei. Myofiber volume was approximated as the volume of a cylinder using the average radius along a 500 µm length of myofiber (n = 6). (**d**) Total isolated RNA levels in gastrocnemius homogenate normalized to muscle mass. (**e**) Z-stack confocal imaging of single myofibers from TA of Pax7-TdTomato mice control, 7 days, 14 days, and 28 days following CLI. Nuclei pseudo-colored in blue, Pax7 in red, actin in green. All scale bars represent 100 µm. Maximum intensity projection performed on all z-stack images (n = 3). (**f**) MuSC frequency of at least 20 single myofibers per sample at various timepoints reported as percentage of Pax7^+^ cells out of total myonuclei (n = 3). ^*^p < 0.05, ^**^p < 0.01, ***p < 0.001, ^****^p < 0.0001 compared to control for all figures. (**g**) Myofiber volume, myonuclei number, myonuclear domain, and MuSC frequency as a ratio of ischemic to control myofiber. Dashed line represents a ratio of 1.

To further elucidate the role of MuSCs, myogenesis, and myonuclear number in the regenerative process of the ischemic myopathy, we examined changes to myonuclear content throughout the entire myofiber after ischemia. To achieve this, single myofibers were isolated (Fig. 3a) and as seen in immunohistochemistry images, ischemic fibers were centrally nucleated. Myonuclei were assembled into longitudinal rows along the center of the myofiber, notably at days 7 and 14, characteristic of newly fused myonuclei derived from differentiated MuSCs^37^. Surprisingly, despite the degeneration of muscle tissue following ischemic injury, a substantial increase in myonuclei number was observed at day 7 that prevails for at least 56 days (Fig. 3b). In addition, we assessed alterations in the myonuclear domain, defined as the cytoplasmic volume of myofiber transcriptionally governed by each myonucleus. By taking z-stack images on a confocal microscope and finding the average radius of the myofiber, the myofiber volume can be approximated as a cylinder. The myonuclear domain is then quantified as the total volume of the myofiber divided by the number of myonuclei within the myofiber. Interestingly, the size of the myonuclear domain was significantly decreased at 7- and 14-days following injury (Fig. 3c), likely as a regenerative response to ischemia in order to enhance transcriptional regulation of the myofiber cytoplasm within its domain, but returns to normal after the tissue perfusion had improved. Consistent with these data, the total amount of RNA isolated from ischemic muscle homogenate was significantly higher at days 7 and 14 compared to control (Fig. 3d).

Since myofibers are multinucleated syncytia formed by the fusion of the differentiated myotubes^8^, we then investigated the frequency of MuSCs following ischemia to ascertain the origin of the accreted myonuclei in the MuSC niche. To explore MuSC frequency, we isolated single myofibers from a tamoxifen-inducible MuSC reporter mouse we generated by crossing *Pax7 Cre*^*ER*^ with *ROSA26-tdTomato* mice (Fig. 3e). As hypothesized, we observed a substantial increase in quiescent Pax7^+^ MuSC content at days 7 and 14 following ischemia, with a peak at day 7 that accompanies the accretion of myonuclei and a concomitant decrease in the myonuclear domain (Fig. 3f). Because Pax7 is a canonical marker for quiescent MuSC^8^, the greater frequency of Pax7^+^ cells implies that MuSCs have undergone adult myogenesis and asymmetric division to increase self-renewal of MuSCs following ischemia to replenish the pool of quiescent MuSCs to continue regenerating and for future rounds of regeneration. Furthermore, the increased number of myonuclei also indicates active differentiation and fusion events of MuSCs into the myofiber following claudication of muscle, further supported by the augmented embryonic myosin heavy chain expression at day 14. The *Pax7-tdTomato* transgenic mouse model also allows detection of MuSCs undergoing myogenesis to fuse with existing myofibers or *de novo* myofiber formation by quantifying the number of tdTomato^+^ myofibers^10^. Indeed, we observed substantial MuSC fusion into myofibers at day 14 following ischemia (Supplemental Fig. S3a,b). Interestingly, all centrally nucleated myofibers were tdTomato^+^, consistent with previous literature that has demonstrated that centrally located myonuclei are derived from differentiated MuSCs^37^. Conversely, to deplete MuSCs before the ischemic injury, we generated a different tamoxifen-inducible mouse model by crossing *Pax7 Cre*^*ER*^ with *ROSA26-diphtheria toxin-A*. Without MuSCs, the soleus muscle, predominantly composed of slow-twitch type I myofibers^33^, was unable to regenerate 28 days following injury (Supplemental Fig. S3c). Taken together (Fig. 3g), these data demonstrate that HLI muscle exhibit considerable MuSC-dependent remodeling of the myonuclear domain to enhance the transcriptional and translation output of the newly formed myofiber and restore muscle mass following injury.

### Mitochondrial network remodels following hindlimb ischemia

**Figure 4:**
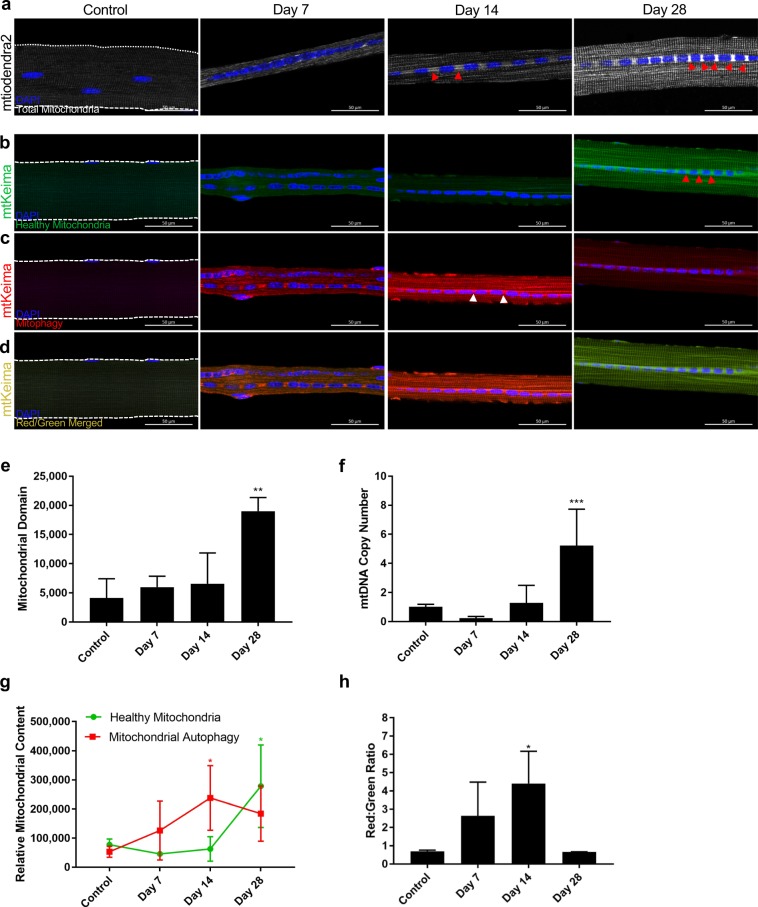
Changes in mitochondrial domain following CLI. (**a**) Confocal imaging of single myofibers from TA of mitoDendra2 mice control, 7 days, 14 days, and 28 days following CLI using constant gain and laser power. Nuclei pseudo-colored in blue, mitochondria in white. Red arrowheads indicate areas of high mitochondrial density. (**b**) Confocal imaging of single myofibers from TA of mtKeima mice in GFP channel to delineate healthy mitochondria. (**c**) Confocal imaging of single myofibers from TA of mtKeima mice in dsRed channel to delineate Mitophagy. (**d**) Merged images of mtKeima myofibers in green (**b**) and red (**c**) channels to portray relative levels of healthy to autophagic mitochondria. Scale bars represent 50 µm. (**e**) Mitochondrial domain of at least 20 single myofibers per sample at various timepoints calculated as relative integrated fluorescent density of total mitochondria in mitoDendra2 mice divided by the number of myonuclei (n = 3). (**f**) Relative mtDNA copy number quantified as expression of mitochondrial-encoded *mt-Co1* normalized by expression of nuclear-encoded *Sdha* (n = 3). (**g**) Relative mitochondrial content of healthy and autophagic mitochondria of at least 20 myofibers per sample in mtKeima mice calculated as mean fluorescent intensity of each fiber (n = 3). (**h**) Red to green ratio of myofibers in mtKeima mice to represent mitochondrial autophagy to healthy mitochondria (n = 3). *p < 0.05, **p < 0.01, ***p < 0.001 compared to control for all figures.

Another critical component of muscle repair is the bioenergetics of regenerating myofibers^38^. Muscle regeneration is a highly energy-dependent process, and an increase in mitochondrial content usually couples with adult myogenesis^39–41^. Moreover, since mitochondrial DNA accounts for only 13 of the proteins that constitute mitochondria while over 99% of mitochondrial proteins are nuclear-encoded^42^, we examined changes in mitochondrial to myonuclear ratio. To this end, we isolated single myofibers from reporter mice expressing a mitochondrial-targeted fluorescent protein (Dendra2)^43^ and imaged the myofibers on a confocal microscope with constant gain and laser power to delineate relative mitochondrial volume of the myofiber (Fig. 4a). In control samples, we noted local mitochondrial networks as punctate and segregated into columns along the Z-line of the myofiber contractile apparatus. At 7 days post-HLI, however, mitochondrial networks in ischemic myofibers spanned several sarcomeres (Fig. 4a)^44^. The reticulum of mitochondria is observed again at 14 and 28 days following HLI. Surprisingly, we found high mitochondrial densities found between the centralized nuclei at later time points, indicated by the red arrowheads (Fig. 4a), suggesting that the new mitochondria are synthesized by the MuSC-derived myonuclei. Because mitochondria are typically compartmentalized to discrete domains centered around their nuclei of origin^44^, we then quantified mitochondrial domain, or relative mitochondrial volume per myonucleus, as a metric to describe changes in the mitochondrial network surrounding each myonucleus. Although there is no change in mitochondrial domain detected at 7- and 14-days following ischemia when the myonuclear domain is at its smallest, we observed an ensuing dramatic increase in mitochondrial domain 28 days following HLI, after myonuclear domain size has returned to normal, but myonuclei number is still elevated (Fig. 4e). Furthermore, to support this striking increase in mitochondrial content, we also quantified relative mtDNA copy number, which correlates with mitochondrial biogenesis^45^. To measure mtDNA copy number^46,47^, we determined the mtDNA/nDNA ratio by quantifying the expression of an mtDNA-encoded mitochondrial gene, *mt-Co1* (Supplemental Fig. S4a), relative to a nuclear-encoded mitochondrial gene, *Sdha* (Supplemental Fig. S4b)^48^. In parallel with the increased mitochondrial volume observed in single myofibers isolated from *mitoDendra2* transgenic mice, mtDNA copy number is significantly upregulated 28 days following ischemic injury (Fig. 4f). This increased mitochondrial content and mtDNA expression at 28 days insinuate greater mitochondrial biogenesis in regenerating myofibers, particularly near the centrally located myonuclei.

To test whether this increase in mitochondria was a compensatory response to prior mitochondrial damage, we employed the mitochondrial-targeted Keima (mtKeima) transgenic mouse model to delineate mitochondrial autophagy or mitophagy. Keima is a pH-dependent fluorescent protein that when targeted to the mitochondria can be used to assess relative quantities of healthy and dysfunctional mitochondria^49^. At the healthy, physiological pH of mitochondria (pH 7.4), the Keima protein exhibits fluorescence with an excitation wavelength of 488 nm and depicts a green signal (Fig. 4b). However, when mitochondria are dysfunctional and selectively degraded by the lysosome through mitophagy, the acidic environment (pH 4.5) shifts the excitation wavelength of the Keima protein to 555 nm to display a red signal (Fig. 4c)^49^. By merging the two channels after confocal imaging of isolated myofibers, relative levels of healthy and autophagic mitochondria within the skeletal muscle after an ischemic injury can be observed by quantifying the mean fluorescent intensity of each signal (Fig. 4d). Consistent with mitoDendra2 data, isolated myofibers from mtKeima mice 14 days following HLI demonstrate high autophagic mitochondrial density between centrally located myonuclei, demarcated by the white arrowheads, implying that the newly synthesized mitochondria are dysfunctional and degraded through mitophagy. However, 28-day samples exhibit increased healthy mitochondrial density between centrally located myonuclei, indicating that most of these newly synthesized mitochondria are functional. Compared to the relative levels of mitophagy and healthy mitochondria in control (red: green ratio), there is a dramatic increase in mitophagy up to 14 days after ischemic injury while the relative number of healthy mitochondria remains unchanged (Fig. 4g,h). However, by day 28, mitophagy decreases, and there is a substantial accumulation of healthy mitochondria in order to restore the physiological ratio of autophagic to healthy mitochondria (Fig. 4g,h). These results suggest high rates of mitochondrial turnover in conjunction with increased mitochondrial biogenesis through 28 days following hindlimb ischemia.

### Mitochondrial membrane is altered following hindlimb ischemia

**Figure 5:**
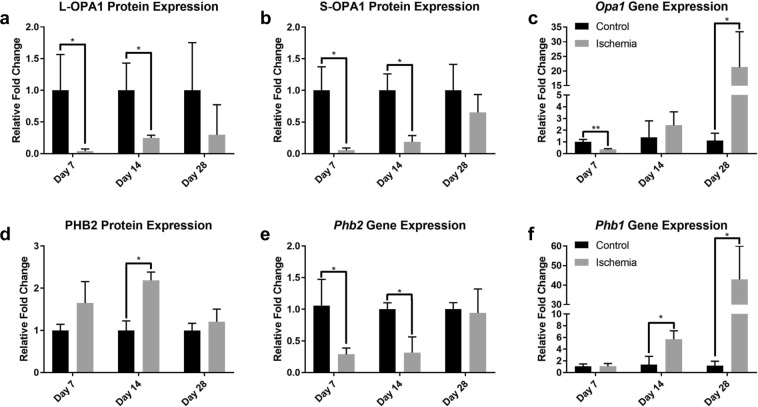
Altered mitochondrial membrane protein expression after CLI. (**a**) Quantified Western blot analyses 7, 14, and 28 days following CLI for the long isoform of OPA1 protein expression (n = 3). (**b**) Quantified Western blot analyses 7, 14, and 28 days following CLI for the short isoform of OPA1 protein expression (n = 3). (**c**) Relative gene expression of *Opa1*7, 14, and 28 days following critical limb ischemia (n = 3). (**d**) Quantified Western blot analyses 7, 14, and 28 days following CLI for prohibitin 2 protein expression (n = 3). (**e**) Relative gene expression of *prohibitin 2* 7, 14, and 28 days following critical limb ischemia (n = 3). (**f**) Relative gene expression of *prohibitin 1*7, 14, and 28 days following critical limb ischemia (n = 3). *p < 0.05, **p < 0.01 compared to control for all figures. Black bars represent control and gray bars represent hindlimb ischemia for all figures.

In order to determine a potential mechanistic link between high mitochondrial turnover and muscle stem cell-derived mitochondrial network remodeling, we next investigated protein expression of OPA1, a nuclear-encoded dynamin-like GTPase that plays an imperative role in the fusion of the inner mitochondrial membrane and stabilization of the cristae ultrastructure^50–52^. An OPA1 precursor containing a mitochondrial targeting sequence is imported into the mitochondrion and proteolytically cleaved into the long (L-OPA1) isoform and anchored to the inner membrane^53^. While L-OPA1 is subsequently processed into the short (S-OPA1) isoform in the matrix and translocated to the intermembrane space^54^, a critical balance of both isoforms is necessary for functional mitochondrial fusion dynamics^55^. Western blot analyses demonstrated considerably diminished protein expression levels of both L-OPA1 (Figs 5a and S5a) and S-OPA1 (Figs 5b and S5b) at 7 and 14 days post-HLI. The decreased OPA1 content of both isoforms at days 7 and 14, coinciding with high levels of mitophagy at these time points, suggests that OPA1 plays a role in the elevated mitochondrial turnover due to impaired mitochondrial dynamics. Interestingly, when *Opa1* gene expression is substantially upregulated by day 28 (Fig. 5c), OPA1 protein levels recover, and the ratio of mitophagy to healthy mitochondria is restored. Furthermore, prohibitin 2 (PHB2), a multi-functional mitochondrial scaffolding protein localized in the inner membrane, is essential for proper cleavage of OPA1 into the L-OPA1 isoform^56^. We observed PHB2 protein significantly elevated at days 14 (Figs 5c and S5c,d), while *Phb2* gene expression is downregulated (Fig. 5e). Since PHB2 import into the mitochondria is dependent on the maintenance of mitochondrial membrane potential, in severely damaged muscle, PHB2 is unable to target depolarized mitochondria and regulate the cleavage of OPA1 appropriately^56^, further suggesting that ischemia induces damage to mitochondria. Intriguingly, prohibitin 1 (PHB1), which is known to modulate mitophagy following oxidative stress^57^, exhibited a striking upregulation in gene expression at 14 and 28 days after ischemic injury (Fig. 5f), indicating that PHB1 may contribute to the functional cleavage of OPA1 by day 28 to compensate PHB2 expression. Overall, these findings indicate that OPA1, PHB2, and PHB1 are key processes that resolve mitochondrial dysfunction and augment mitochondrial dynamics following HLI.

### Mitochondrial function is impaired after hindlimb ischemia

**Figure 6:**
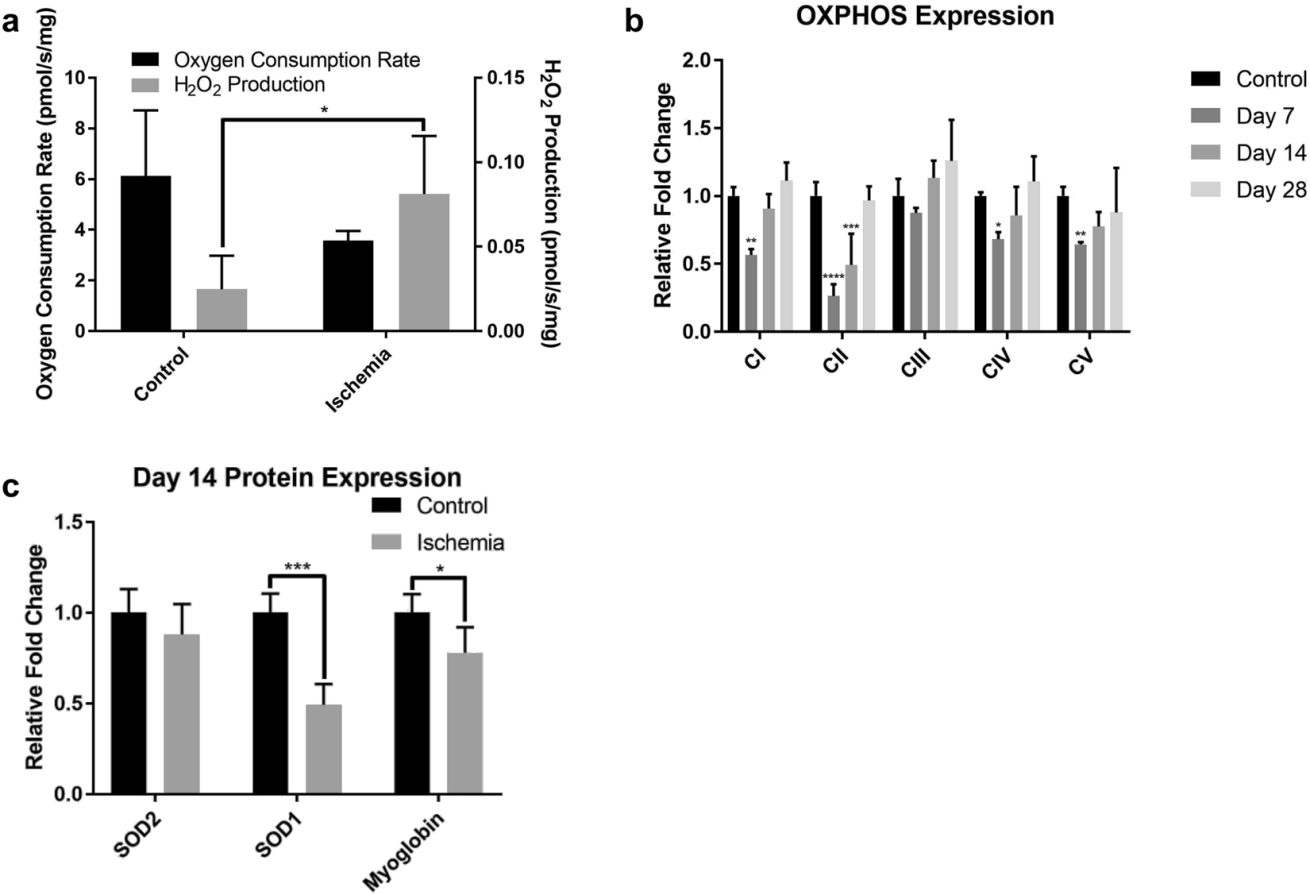
Impaired mitochondrial function following CLI. (**a**) Basal (state 1 respiration) oxygen consumption rate and mitochondrial hydrogen peroxide (H_2_O_2_) production from hindlimb skeletal muscles 7 days following CLI using Oroboros Oxygraph-2k (n = 3). (**b**) Quantified Western blot analyses 7, 14, and 28 days following CLI for mitochondrial ETC complex I (NDUFB8-subunit), complex II (SDHB-subunit), complex III (UQCRC2-subunit), complex IV (MTCO1-subunit), and complex V (ATP5A-subunit) relative protein expression (n = 3). (**c**) Quantified Western blot analyses 14 days following CLI for SOD2 (n = 3), SOD1 (n = 6), and myoglobin (n = 6) protein expression. *p < 0.05, **p < 0.01, ***p < 0.001, ****p < 0.0001 compared to control for all figures.

Because mitochondrial dynamics are regulated by redox state^58^, we next investigated alterations to mitochondrial oxidative phosphorylation and ROS generation by performing functional analyses and measuring levels of various proteins associated with mitochondria at days 7, 14, and 28. We analyzed the bioenergetic function of isolated mitochondria at day 7 by measuring basal (State 1) respiration. The basal oxygen consumption did not reach a statistical difference. However, in State 1, mitochondrial emission of H_2_O_2_ was ~3-fold higher in ischemic muscle compared to contralateral control (Fig. 6a)^59^. To further assess mitochondrial function, Western blot analyses (Fig. 6b and Supplemental Fig. S5e,f) for the electron transport chain complexes also revealed that subunits of complexes I, II, and IV at day 7 and complex II at day 14 were significantly declined, suggesting a potential disruption in mitochondrial bioenergetics and concomitant release of ROS following ischemia and reperfusion (Fig. 6a,b). However, by day 28, the expression of all electron transport chain complexes returned to control (Fig. 6b). Furthermore, the protein levels of key antioxidant enzymes, SOD2 (MnSOD) in the mitochondrial matrix and SOD1 (CuZnSOD) in the intermembrane space and cytosol, exhibited no change in SOD2 content, but a significant decrease in SOD1 content on day 14 (Fig. 6c). Finally, the amount of the oxygen-binding protein myoglobin, typically correlated with mitochondria content^60^, was decreased at day 14, supporting the loss of myoglobin-rich type I fibers following ischemia and suggesting decreased oxygen utilization and high mitochondrial turnover in the ischemic muscle (Fig. 6c). Taken together, these results demonstrate a novel finding that an ischemia-induced mitochondrial dysfunction is strongly coupled with MuSC-induced mitochondrial domain remodeling and mitochondrial biogenesis to restore skeletal muscle bioenergetic function in the newly regenerated myofibers.

## Discussion

In the present study, we report hindlimb ischemia-induced remodeling of various niche components of the MuSC, particularly the neuromuscular junction of the motor unit, myonuclear number, and mitochondrial content of the myofiber. Due to the delayed regenerative response of skeletal muscle as a result of the abnormal perfusion to the tissues, we observed incomplete regeneration of the MuSC niche components for up to 56 days. Specifically, myofibers were still centrally nucleated while NMJs were incompletely reinnervated for at least 56 days. Ischemia-induced denervation at early time points resulted in a subsequent reorganization of the motor units that were associated with a loss of slow-twitch, type I fibers at days 14 and 28. Moreover, the increase in subsynaptic nuclei that maintain the NMJ synaptic transmission following ischemia supports the notion that MuSCs play a role in the repair of the NMJ^24^. In parallel, the MuSC-mediated increase in number of the myonuclei within a muscle fiber and its concomitant decrease in myonuclear domain, or cytoplasmic volume governed by each myonucleus, are likely a part of the repair mechanism of skeletal muscle in order to restore the transcriptional and translational output of contractile and structural components of regenerating myofibers. The smaller myonuclear domain following an ischemia-induced fusion of MuSCs into damaged fibers also allows enhanced expression of transcripts required for mitochondrial and myofibrillar network remodeling. We also demonstrate for the first time that ischemia-reperfusion-induced changes in the myonuclear remodeling are significantly correlated with the mitochondrial content adjacent to centrally located myonuclei, known as the mitochondrial domain, to potentially support the high energy demands of the regenerating tissues and remodel the mitochondrial reticulum of the myofibers.

In order to gain a more comprehensive understanding of mitochondrial biogenesis during ischemia/reperfusion-induced skeletal muscle regeneration, it is necessary to determine the origin of the accumulated mitochondria. While 13 proteins of the mitochondrial electron transport chain are encoded by mtDNA, thousands of mitochondrial proteins are nuclear-encoded and need to be imported into the mitochondria^42^, illustrated by the high mitochondrial density localized adjacent to myonuclei. For example, a scaffold protein known as OPA1, which is encoded in the nuclear genome but contains a mitochondrial targeting sequence, maintains the cristae organization and plays a major role in mitochondrial dynamics by regulating fusion of the inner membrane^50–52^. Based on the findings from this study, there are several possible explanations for the increase in mitochondrial content. First, oxidative stress and ROS may directly upregulate the genes required for mitochondrial dynamics (fusion and fission)^61^. Because mitochondrial fission of damaged fragments is a requirement of mitochondrial autophagy^62^, there may be an increase of fission events in myofibers following hindlimb ischemia. However, the diminished levels of OPA1, required for mitochondrial fusion^50–52^, and the sensitivity of OPA1 to high levels of ROS generated after an ischemic injury^58^ indicate that mitochondrial fusion is debilitated. Therefore, it is unlikely that mitochondrial dynamics dictate increased mitochondrial content.

A second possible explanation for the increased mitochondrial content is because of the mitochondrial dysfunction and impaired bioenergetics^63^, myonuclei may respond as an adaptive measure to upregulate expression of the genes required to produce functional mitochondria and meet the high energy demands of skeletal muscle. This is accomplished by MuSCs fusing with degenerating myofibers to increase the number of myonuclei that can more generate transcripts for the biogenesis of the surrounding mitochondria. While we observe an increase in quiescent Pax7^+^ MuSCs that have few active mitochondria and rely on glycolytic metabolism at day 7 following injury^64^, this is accompanied by an increase in differentiating myogenic progenies through the asymmetric division of MuSCs that can fuse into multinucleated myotubes^65^. The myotubes then undergo a late fusion into regenerating myofibers to produce mature myonuclei that migrate to the center of the myofiber^37^, supported by the accretion of myonuclei and their locations after an ischemic insult. These MuSC-derived myonuclei may also produce genes such as SIRT1, PGC-1α, FOXO3, or AMPK in order to initiate mitochondrial biogenesis^66–68^. Due to the ischemia-induced damage to the mitochondria, it is likely that these myonuclei synthesize new mitochondria to remodel the mitochondrial network within its domain. To corroborate this hypothesis, our findings display remarkably high mitochondrial densities located between the centrally positioned myonuclei as a mechanism to reset its mitochondrial domain. Proteins such as the prohibitin isoforms, which have been shown to possess both the mitochondrial- and nuclear-targeting sequence to assist in the import of nuclear-encoded mitochondrial proteins such as OPA1^56,69,70^, allow the mitochondrial genome and the nuclear genome to communicate during muscle regeneration. Signified by the elevated levels of prohibitin 2 and the close proximity of mitochondria to the myonuclei following ischemia, we suggest for the first time that MuSC-derived myonuclei drive biogenesis of mitochondria to restore the compartmentalized architecture of the mitochondrial reticula within a regenerating myofiber. To further elucidate the mechanism behind the mitochondrial biogenesis, we plan to examine the roles of master regulators involved in mitochondrial formation and dynamics and conduct a loss-of-function study by analyzing myonuclei number and mitochondrial content in transgenic mice with depleted MuSCs.

While we establish a notable increase in mitochondrial content by day 28 following ischemia, our data also contributes insight into the preceding ischemia-induced mitochondrial dysfunction. While ROS, such as hydrogen peroxide (H_2_O_2_), is required for redox signaling and regulation of transcription factors, imbalanced homeostasis resulting in excess H_2_O_2_ production can oxidize lipids, proteins, and nucleic acids of the MuSC niche^71^. Mitochondria are one of the major sources of ROS and generate H_2_O_2_ by converting the highly reactive superoxide into the more stable H_2_O_2_, catalyzed by the antioxidant enzyme superoxide dismutase (SOD). To elucidate the precise origin of increased H_2_O_2_ production in ischemic mitochondria, the enzymatic activity of the mitochondrial membrane complexes and isoforms of SOD as well as quantification of superoxide levels are required. Although complex I releases ROS into the mitochondrial matrix, complex III releases ROS to both sides of the inner membrane^72^. Since the content of SOD2 (MnSOD), primarily localized in the matrix, is unchanged while the contents of SOD1 (CuZnSOD) and OPA1, both found in the intermembrane space and along the inner membrane, are decreased, we postulate that ROS are present in the intermembrane space due to complex III-mediated electron leak. So as to fully characterize the mitochondrial dysfunction following ischemia, we plan to analyze complex III activity to test this hypothesis.

Finally, because the vasculature is required in biological tissues to supply key nutrients, growth factors, chemokines, oxygen, and immune cells that initiate tissue regeneration and regulate mitochondrial function^11–14,73^, it is evident that functional perfusion to the tissues is a key determinant in regeneration kinetics. It is noteworthy, however, that our studies were conducted on *C57BL/6J* mice, which have been shown to exhibit more pre-existing collateral vessels^74^, greater angiogenic potential, higher revascularization rates^75^, increased mitochondrial respiration^76^, and enhanced skeletal muscle regeneration compared to other strains of mice (i.e., *BALB/c*, *129S2/Sv*)^77^. While beyond the scope of this current study, similar genetic polymorphisms may manifest in the population of human CLI patients and thus, it is worthwhile to study the effects of ischemia in various mouse strains to provide a more representative animal model of CLI despite genetic differences.

In summary, we demonstrate remodeling of MuSC niche components following ischemic injury, notably through neuromuscular junction repair by subsynaptic nuclei, increased myonuclei number, decreased myonuclear domain, and greater mitochondria content per nucleus of skeletal muscle fibers. These data also indicate that CLI resets the myonuclear and mitochondrial domains, coordinated by the muscle stem cell, as part of the regenerative mechanism in ischemic conditions. The findings from this study illustrate the complex regenerative response of the MuSC niche following critical limb ischemia and serve as a basis to further explore mechanisms of mitochondrial biogenesis in skeletal muscle regeneration.

## Methods

### Animal models

All animal procedures were conducted under the approval of the Institutional Animal Care and Use Committee (IACUC) of the Georgia Institute of Technology and performed in accordance with all relevant guidelines and regulations. All mice in this study were either *C57BL/6J* genetic background or backcrossed with *C57BL/6J* for more than 6 generations and were initially purchased from Jackson Laboratory. Mice were bred and maintained in pathogen-free conditions with a 12–12 light/dark cycle in the Physiological Research Laboratory (PRL) at Georgia Institute of Technology. For muscle stem/satellite cell reporter, mice expressing a tamoxifen-inducible *Cre* from the endogenous Pax7 promoter were bred with mice carrying a loxP-flanked STOP cassette followed by TdTomato in the ROSA26 locus. Alternatively, to deplete muscle stem cells, mice expressing a tamoxifen-inducible *Cre* from the *Pax7* promoter were bred with mice carrying a loxP-flanked STOP cassette followed by diphtheria toxin-A (DTA) in the ROSA26 locus. Other transgenic reporter mice include: *Thy1-EYFP (line 16)* for motor neuron^32^, *mitoDendra2* for mitochondria^43,44^, *mtKeima* for mitochondrial autophagy^49,78^, and *PV Cre*^*ER*^*; ChR2-EYFP* (tamoxifen-inducible Cre recombinase inserted into PV locus bred with mice carrying a loxP-flanked STOP cassette followed by ChR2-EYFP) for expression of EYFP driven by calcium-binding protein parvalbumin to detect type IIB and IIX fast-twitch fibers^79^. Both males and females aged between 3 and 6 months, considered young adults, were used in a randomized manner for all experiments in this study. No differences in muscle regeneration were observed in response to ischemic injury between 3-month old and 6-month old mice.

### Surgical procedure

To study the effects of critical limb ischemia, we employed a well-characterized murine hindlimb ischemia (HLI) surgical ligation model^80^. Briefly, a small unilateral incision (1 cm long) was made from the ankle to the medial thigh to expose the femoral vessels. The femoral artery and vein were then ligated with 5–0 sutures between the superficial epigastric artery and profunda femoris artery. A second ligation was made proximal to the branching of the tibial arteries, and the segment of vessels between the two ligations was excised. The skin was then closed with both sutures and wound clips. The sham surgery, where the femoral artery and vein were exposed similar to the method above without ligation or excision, was performed on the contralateral leg. Animals were maintained in single-housed cages for 3–56 days following HLI. Laser Doppler Perfusion Imaging (LDPI) was performed on a MoorLDI Imager at a scan resolution of 210 × 160 pixels and a height of 21 cm before euthanization by CO_2_ inhalation. Perfusion was quantified in the lower hindlimb using MoorLDI Software V5.3.

### Histochemistry and immunostaining

Immediately following euthanization of animals, the tibialis anterior (TA) muscles were either snap frozen in 2-methylbutane cooled by liquid nitrogen for cryo-sectioning or fixed in 4% paraformaldehyde for single myofiber isolation. Frozen TA was sliced into 10 µm sections while fixed TA was mechanically separated into 20–30 single myofibers per sample from random areas of the muscle. The gastrocnemius was frozen in liquid nitrogen for Western blot analysis while the extensor digitorum longus (EDL) was fixed for immunostaining. Hematoxylin and eosin (H&E) and immunofluorescence were performed as previously described^81^. Materials and antibodies can be found in Supplemental Table 1 for dilution factors, vendors, and catalog numbers of materials. All images were taken on either Zeiss Axio Observer D1 or Zeiss 700 Laser Scanning Confocal microscopes and quantified using ImageJ. Z-stack images were taken to obtain a 3D rendering of myofibers in ImageJ and myofiber volume was approximated as the volume of a cylinder using the average radius along a 500 µm length of the myofiber.

### Western blot analysis

Gastrocnemii were homogenized with 25 strokes using a 5 mL PTFE tissue grinder with clearance 0.15–0.25 mm (VWR 89026-392, 89026-404) at 3,000 rpm in RIPA lysis buffer (VWR 97063-270) supplemented with Roche cOmplete Mini Protease Inhibitors (Roche 04693124001) and PhosSTOP Phosphatase Inhibitors (Roche 04906837001). Following 3 freeze-thaw cycles in liquid nitrogen and on ice, the samples were centrifuged at 18,400 *g* for 10 minutes, and the supernatants (homogenates) were normalized to total protein concentration using a BCA protein assay kit (Thermo 23225). 50 µg of protein were run through 4–20% Criterion TGX Gels (Bio-Rad 5671093) at 150 V for 185 minutes and transferred to a PVDF membrane using a Trans-Blot Turbo System at 2.5 A for 7 minutes. Ponceau staining (Sigma P7170) was used as a loading control. Antibodies used can be found in Supplementary Table 1. Membranes were imaged on Li-Cor Odyssey CLx-1050 Infrared Imaging System and bands were quantified on Li-Cor Image Studio V5.2.

### RNA Isolation and quantitative polymerase chain reaction

Using the normalized tissue homogenate prepared for western blot analysis, 50 µL of homogenate was added to 300 µL of RLT buffer supplied in the RNeasy® Mini Kit (Qiagen 74104) supplemented with 1% β-mercaptoethanol to inactivate RNases. The protocol according to Qiagen RNeasy® kit was followed for the remaining RNA isolation steps. The RNA concentration was measured by a NanoDrop One while A260/230 and A260/280 ratios were calculated for quality control. RNA content was then calculated by normalizing the RNA concentration to total muscle mass. To reverse transcribe the RNA to copy DNA, RNA concentrations were normalized to each other and the protocol according to Applied Biosystems High-Capacity cDNA Reverse Transcription Kit (Applied Biosystems 4368814) was followed, and the samples were run in a thermal cycler according to the recommended conditions. Finally, the Applied Biosystems PowerUp SYBR Green Master Mix (Applied Biosystems A25742) was used with the primers found in Supplementary Table S4 in an Applied Biosystems StepOnePlus Real-Time PCR system to perform the qPCR reactions. β-actin and B2M, which were found to be stably expressed following ischemia, were used housekeeping genes to quantify relative fold induction.

### Mitochondria functional testing

Mitochondria were isolated from lower limb muscles distal to the knee by differential centrifugation. Muscles from each leg were digested with 5 mL dispase II (100 µg/mL) and trypsin (1 mg/mL) in Chappel-Perry buffer (see Supplementary Table S2 for detail). The digested tissue was then homogenized, centrifuged at 12,000 *g* for 10 minutes, and resuspended in 5 mL Chappel-Perry buffer II (Supplementary Table S2). The suspensions were then centrifuged at 600 *g* for 10 minutes, and the supernatants subsequently centrifuged at 7,000 *g* to pellet the mitochondria. 300 µg of mitochondria resuspended in respiration buffer (Supplementary Table S2) was used immediately in the Oroboros Oxygraph-2k high-resolution respirometer for basal (state 1) oxygen consumption rate and H_2_O_2_ generation detected with the Amplex Red assay kit.

### Statistical analysis

All statistical analyses in this study were performed on GraphPad Prism 7 and data is presented as mean ± standard deviation (SD). Data from the contralateral controls did not change over time; therefore, multiple comparison tests for significance were performed between the mean of control and each time point. Normality of data was tested with the Shapiro-Wilk test. For experiments in which data was collected from different mice over time, a one-way ANOVA with Tukey’s *post hoc* test or Kruskal-Wallis test with Dunn’s *post hoc* test were used based on normality. A paired two-tailed t-test or Mann-Whitney U test was used to compare the injured hindlimbs to their contralateral controls based on normality. A two-way ANOVA with Tukey’s *post hoc* test was performed for experiments with various time points where each ischemic sample was compared to its contralateral control. A *p*-value of less than 0.05 was considered statistically significant.

## Supplementary information

Supplemental Information

## Data Availability

The datasets generated during and/or analyzed during the current study are available from the corresponding author on reasonable request.

## References

[1] Swaminathan A, Vemulapalli S, Patel MR, Jones WS (2014). Lower extremity amputation in peripheral artery disease: improving patient outcomes. Vasc Health Risk Manag.

[2] Olea FD (2015). Vascular endothelial growth factor overexpression does not enhance adipose stromal cell-induced protection on muscle damage in critical limb ischemia. Arteriosclerosis, thrombosis, and vascular biology.

[3] Cooke JP, Losordo DW (2015). Modulating the Vascular Response to Limb Ischemia Angiogenic and Cell Therapies. Circulation research.

[4] Borselli C (2010). Functional muscle regeneration with combined delivery of angiogenesis and myogenesis factors. Proceedings of the National Academy of Sciences of the United States of America.

[5] Brooks PC (1996). Role of integrins in angiogenesis. European Journal of Cancer.

[6] Kilarski Witold W, Samolov Branka, Petersson Ludvig, Kvanta Anders, Gerwins Pär (2009). Biomechanical regulation of blood vessel growth during tissue vascularization. Nature Medicine.

[7] Charge SB, Rudnicki MA (2004). Cellular and molecular regulation of muscle regeneration. Physiological reviews.

[8] Hart CA, Tsui J, Khanna A, Abraham DJ, Baker DM (2013). Stem cells of the lower limb: their role and potential in management of critical limb ischemia. Experimental biology and medicine (Maywood, N.J.).

[9] Yin H, Price F, Rudnicki MA (2013). Satellite Cells and the Muscle Stem Cell Niche. Physiological Reviews.

[10] Verma M (2018). Muscle Satellite Cell Cross-Talk with a Vascular Niche Maintains Quiescence via VEGF and Notch Signaling. Cell Stem Cell.

[11] Mandal S, Lindgren AG, Srivastava AS, Clark AT, Banerjee U (2011). Mitochondrial function controls proliferation and early differentiation potential of embryonic stem cells. Stem cells (Dayton, Ohio).

[12] Richter C (1995). Oxidants in mitochondria: from physiology to diseases. Biochimica et Biophysica Acta (BBA) - Molecular Basis of Disease.

[13] Latroche C (2015). Skeletal Muscle Microvasculature: A Highly Dynamic Lifeline. Physiology.

[14] Sciorati C, Rigamonti E, Manfredi AA, Rovere-Querini P (2016). Cell death, clearance and immunity in the skeletal muscle. Cell Death and Differentiation.

[15] Vignaud A (2010). Impaired Skeletal Muscle Repair after Ischemia-Reperfusion Injury in Mice. Journal of Biomedicine and Biotechnology.

[16] Yang X (2001). Patterning of Muscle Acetylcholine Receptor Gene Expression in the Absence of Motor Innervation. Neuron.

[17] Rowan SL (2012). Denervation Causes Fiber Atrophy and Myosin Heavy Chain Co-Expression in Senescent Skeletal Muscle. PLOS ONE.

[18] Holloszy JO, Larsson L (1995). Motor Units: Remodeling in Aged. Animals. The Journals of Gerontology: Series A.

[19] Martin P, Lewis J (1989). Origins of the neurovascular bundle: interactions between developing nerves and blood vessels in embryonic chick skin. The International journal of developmental biology.

[20] Eichmann A, Makinen T, Alitalo K (2005). Neural guidance molecules regulate vascular remodeling and vessel navigation. Genes & development.

[21] Cattin A-L (2015). Macrophage-Induced Blood Vessels Guide Schwann Cell-Mediated Regeneration of Peripheral Nerves. Cell.

[22] Shvartsman D (2014). Sustained Delivery of VEGF Maintains Innervation and Promotes Reperfusion in Ischemic Skeletal Muscles Via NGF/GDNF Signaling. Molecular Therapy.

[23] Wilson RJ (2018). Mitochondrial protein S-nitrosation protects against ischemia reperfusion-induced denervation at neuromuscular junction in skeletal muscle. Free radical biology & medicine.

[24] Schultz E (1978). Changes in the satellite cells of growing muscle following denervation. The Anatomical record.

[25] Liu, W., Wei-LaPierre, L., Klose, A., Dirksen, R. T. & Chakkalakal, J. V. Inducible depletion of adult skeletal muscle stem cells impairs the regeneration of neuromuscular junctions. *Elife* **4**, 10.7554/eLife.09221 (2015). 10.7554/eLife.09221 PMC4579298 26312504

[26] Alnaes E, Rahamimoff R (1975). On the role of mitochondria in transmitter release from motor nerve terminals. The Journal of physiology.

[27] Verstreken P (2005). Synaptic mitochondria are critical for mobilization of reserve pool vesicles at Drosophila neuromuscular junctions. Neuron.

[28] Karsch-Mizrachi I, Travis M, Blau H, Leinwand LA (1989). Expression and DNA sequence analysis of a human embryonic skeletal muscle myosin heavy chain gene. Nucleic acids research.

[29] Mahdy MAA, Lei HY, Wakamatsu J-I, Hosaka YZ, Nishimura T (2015). Comparative study of muscle regeneration following cardiotoxin and glycerol injury. Annals of Anatomy - Anatomischer Anzeiger.

[30] Hardy D (2016). Comparative Study of Injury Models for Studying Muscle Regeneration in Mice. PLoS One.

[31] Tintignac LA, Brenner H-R, Rüegg MA (2015). Mechanisms Regulating Neuromuscular Junction Development and Function and Causes of Muscle Wasting. Physiological Reviews.

[32] Feng G (2000). Imaging neuronal subsets in transgenic mice expressing multiple spectral variants of GFP. Neuron.

[33] Burkholder TJ, Fingado B, Baron S, Lieber RL (1994). Relationship between muscle fiber types and sizes and muscle architectural properties in the mouse hindlimb. Journal of Morphology.

[34] Buller AJ, Eccles JC, Eccles RM (1960). Interactions between motoneurones and muscles in respect of the characteristic speeds of their responses. The Journal of physiology.

[35] Simon AM, Hoppe P, Burden SJ (1992). Spatial restriction of AChR gene expression to subsynaptic nuclei. Development (Cambridge, England).

[36] Jang YC (2010). Increased superoxide *in vivo* accelerates age-associated muscle atrophy through mitochondrial dysfunction and neuromuscular junction degeneration. The FASEB Journal.

[37] Cadot B (2012). Nuclear movement during myotube formation is microtubule and dynein dependent and is regulated by Cdc42, Par6 and Par3. EMBO reports.

[38] Rochard P (2000). Mitochondrial activity is involved in the regulation of myoblast differentiation through myogenin expression and activity of myogenic factors. The Journal of biological chemistry.

[39] Moyes CD, Mathieu-Costello OA, Tsuchiya N, Filburn C, Hansford RG (1997). Mitochondrial biogenesis during cellular differentiation. The American journal of physiology.

[40] Remels AH (2010). Regulation of mitochondrial biogenesis during myogenesis. Molecular and cellular endocrinology.

[41] Duguez S, Feasson L, Denis C, Freyssenet D (2002). Mitochondrial biogenesis during skeletal muscle regeneration. American journal of physiology. Endocrinology and metabolism.

[42] Boengler K, Heusch G, Schulz R (2011). Nuclear-encoded mitochondrial proteins and their role in cardioprotection. Biochimica et biophysica acta.

[43] Pham AH, McCaffery JM, Chan DC (2012). Mouse lines with photo-activatable mitochondria to study mitochondrial dynamics. Genesis.

[44] Mishra P, Varuzhanyan G, Pham AH, Chan DC (2015). Mitochondrial dynamics is a distinguishing feature of skeletal muscle fiber types and regulates organellar compartmentalization. Cell metabolism.

[45] Wu H (2002). Regulation of mitochondrial biogenesis in skeletal muscle by CaMK. Science (New York, N.Y.).

[46] Quiros PM, Goyal A, Jha P, Auwerx J (2017). Analysis of mtDNA/nDNA Ratio in Mice. Current protocols in mouse biology.

[47] Rooney JP (2015). PCR based determination of mitochondrial DNA copy number in multiple species. Methods in molecular biology (Clifton, N.J.).

[48] Taanman J-W (1999). The mitochondrial genome: structure, transcription, translation and replication. Biochimica et Biophysica Acta (BBA) - Bioenergetics.

[49] Sun N (2017). A fluorescence-based imaging method to measure *in vitro* and *in vivo* mitophagy using mt-Keima. Nature protocols.

[50] Meeusen S (2006). Mitochondrial inner-membrane fusion and crista maintenance requires the dynamin-related GTPase Mgm1. Cell.

[51] Song Z, Ghochani M, McCaffery JM, Frey TG, Chan DC (2009). Mitofusins and OPA1 mediate sequential steps in mitochondrial membrane fusion. Molecular biology of the cell.

[52] Frezza C (2006). OPA1 controls apoptotic cristae remodeling independently from mitochondrial fusion. Cell.

[53] Ishihara N, Fujita Y, Oka T, Mihara K (2006). Regulation of mitochondrial morphology through proteolytic cleavage of OPA1. The EMBO journal.

[54] DeVay RM (2009). Coassembly of Mgm1 isoforms requires cardiolipin and mediates mitochondrial inner membrane fusion. The Journal of cell biology.

[55] Song Z, Chen H, Fiket M, Alexander C, Chan DC (2007). OPA1 processing controls mitochondrial fusion and is regulated by mRNA splicing, membrane potential, and Yme1L. The Journal of cell biology.

[56] Merkwirth C (2008). Prohibitins control cell proliferation and apoptosis by regulating OPA1-dependent cristae morphogenesis in mitochondria. Genes & development.

[57] Kathiria AS (2012). Prohibitin 1 Modulates Mitochondrial Stress-Related Autophagy in Human Colonic Epithelial Cells. PLOS ONE.

[58] Norton M (2014). ROMO1 is an essential redox-dependent regulator of mitochondrial dynamics. Science signaling.

[59] Pipinos II (2006). Mitochondrial defects and oxidative damage in patients with peripheral arterial disease. Free radical biology & medicine.

[60] Yamada T (2013). Interaction between myoglobin and mitochondria in rat skeletal muscle. Journal of applied physiology (Bethesda, Md.: 1985).

[61] Miranda S, Foncea R, Guerrero J, Leighton F (1999). Oxidative Stress and Upregulation of Mitochondrial Biogenesis Genes in Mitochondrial DNA-Depleted HeLa Cells. Biochemical and Biophysical Research Communications.

[62] Twig G (2008). Fission and selective fusion govern mitochondrial segregation and elimination by autophagy. The EMBO journal.

[63] Pipinos II (2003). Abnormal mitochondrial respiration in skeletal muscle in patients with peripheral arterial disease. Journal of Vascular Surgery.

[64] Liu W (2012). Hypoxia promotes satellite cell self-renewal and enhances the efficiency of myoblast transplantation. Development (Cambridge, England).

[65] Berendse M, Grounds MD, Lloyd CM (2003). Myoblast structure affects subsequent skeletal myotube morphology and sarcomere assembly. Experimental cell research.

[66] LeBleu VS (2014). PGC-1alpha mediates mitochondrial biogenesis and oxidative phosphorylation in cancer cells to promote metastasis. Nat Cell Biol.

[67] Jager S, Handschin C, St-Pierre J, Spiegelman BM (2007). AMP-activated protein kinase (AMPK) action in skeletal muscle via direct phosphorylation of PGC-1alpha. Proceedings of the National Academy of Sciences of the United States of America.

[68] Canto C (2009). AMPK regulates energy expenditure by modulating NAD+ metabolism and SIRT1 activity. Nature.

[69] Fusaro G, Dasgupta P, Rastogi S, Joshi B, Chellappan S (2003). Prohibitin induces the transcriptional activity of p53 and is exported from the nucleus upon apoptotic signaling. The Journal of biological chemistry.

[70] Kasashima K, Ohta E, Kagawa Y, Endo H (2006). Mitochondrial functions and estrogen receptor-dependent nuclear translocation of pleiotropic human prohibitin 2. The Journal of biological chemistry.

[71] Marinho HS, Real C, Cyrne L, Soares H, Antunes F (2014). Hydrogen peroxide sensing, signaling and regulation of transcription factors. Redox Biology.

[72] Muller, F. L., Liu, Y. & Van Remmen, H. Complex III releases superoxide to both sides of the inner mitochondrial membrane. *Journal of Biological Chemistry* (2004). 10.1074/jbc.M407715200 15317809

[73] Ryan, T. E. *et al*. Mitochondrial Regulation of the Muscle Microenvironment in Critical Limb Ischemia. *Frontiers in Physiology* **6**, 10.3389/fphys.2015.00336 (2015). 10.3389/fphys.2015.00336 PMC4649016 26635622

[74] Helisch A (2006). Impact of mouse strain differences in innate hindlimb collateral vasculature. Arteriosclerosis, thrombosis, and vascular biology.

[75] McClung JM (2012). Skeletal muscle-specific genetic determinants contribute to the differential strain-dependent effects of hindlimb ischemia in mice. Am J Pathol.

[76] Schmidt CA (2017). Diminished force production and mitochondrial respiratory deficits are strain-dependent myopathies of subacute limb ischemia. J Vasc Surg.

[77] Grounds MD, McGeachie JK (1989). A comparison of muscle precursor replication in crush-injured skeletal muscle of Swiss and BALBc mice. Cell and tissue research.

[78] Sun N (2015). Measuring *In Vivo* Mitophagy. Molecular Cell.

[79] Chakkalakal JV, Kuang S, Buffelli M, Lichtman JW, Sanes JR (2012). Mouse transgenic lines that selectively label type I, type IIa, and types IIX + B skeletal muscle fibers. genesis.

[80] Couffinhal T (1998). Mouse model of angiogenesis. The American Journal of Pathology.

[81] Anderson SE (2019). Determination of a Critical Size Threshold for Volumetric Muscle Loss in the Mouse Quadriceps. Tissue Engineering Part C: Methods.

