# Supplementary material for: Critical Limb Ischemia Induces Remodeling of Skeletal Muscle Motor Unit, Myonuclear-, and Mitochondrial-Domains
Source: Sci Rep. 2019 Jul 2;9:9551. doi: 10.1038/s41598-019-45923-4 (PMC6606576; doi:10.1038/s41598-019-45923-4)
Supplement: Supplementary file 1 — Supplemental Information [file 41598_2019_45923_MOESM1_ESM.docx]

**Supplemental Information**

**Critical Limb Ischemia Induces Remodeling of Skeletal Muscle Motor Unit, Myonuclear-, and Mitochondrial-Domains**

Mahir Mohiuddin, Nan Hee Lee, June Young Moon, Woojin M. Han, Shannon E. Anderson, Jeongmoon J. Choi, Eunjung Shin, Shadi A. Nakhai, Thu Tran, Berna Aliya, Do Young Kim, Aimee Gerold, Laura M. Hansen, W. Robert Taylor, and Young C. Jang

**
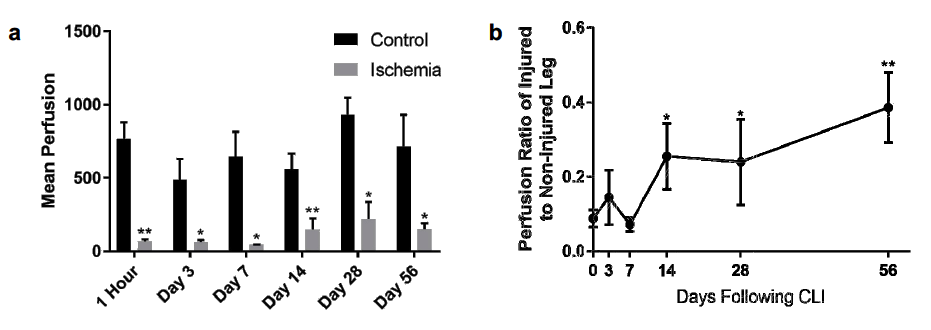
**

**Supplementary Figure S1. Quantification of Laser Doppler Perfusion Imaging.** (a) Mean perfusion to hindlimb distal to the knee in ischemic leg and contralateral control over 56 days. n=6, **p*<0.05, ***p*<0.01 using two-way ANOVA with Tukey’s *post hoc* test. (b) Ratio of perfusion in ischemic leg to control over 56 days. n=6, **p*<0.05, ***p*<0.01 compared to day 0 using one-way ANOVA with Tukey’s *post hoc* test.

**
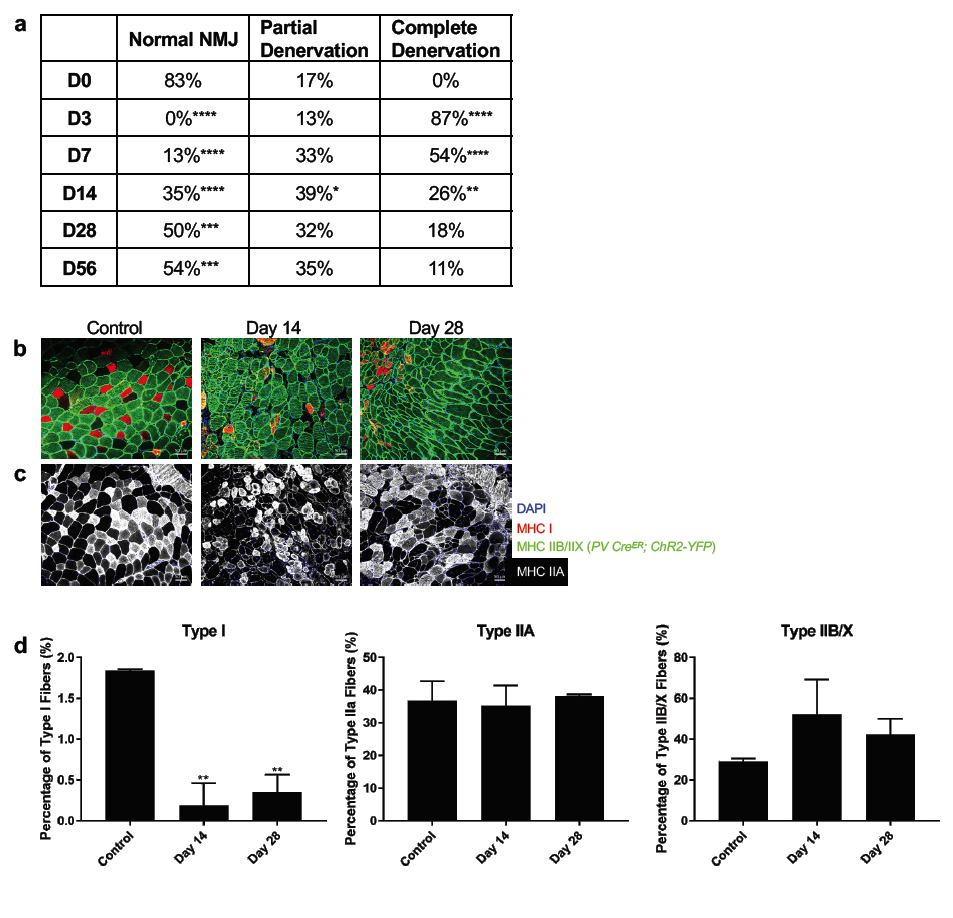
**

**Supplementary Figure S2. NMJ Categorization and Fiber Type Shifting.** (a) Average data values of NMJ denervation states as shown in Fig. 2c with significant differences compared to day 0 denoted by asterisks. (b) TA cross-sections of *PV-Cre; ChR2-YFP* mice for expression of fiber type IIB/IIX (MHC IIB/IIX) and stained for fiber type I (MHC I) in control, 14 days, and 28 days following HLI. Nuclei pseudo-colored in blue, MHC I in red, and MHC IIB/IIX in green. (c) TA cross-sections of wildtype mice stained for fiber type IIA (MHC IIA) in control, 14 days, and 28 days following HLI. Nuclei pseudo-colored in blue, MHC IIA in white. Scale bars represent 50 µm. (d) Percentages of type I, IIA, and IIB/X fibers, respectively. n=3, ^**^*p*<0.01 compared to control.

**
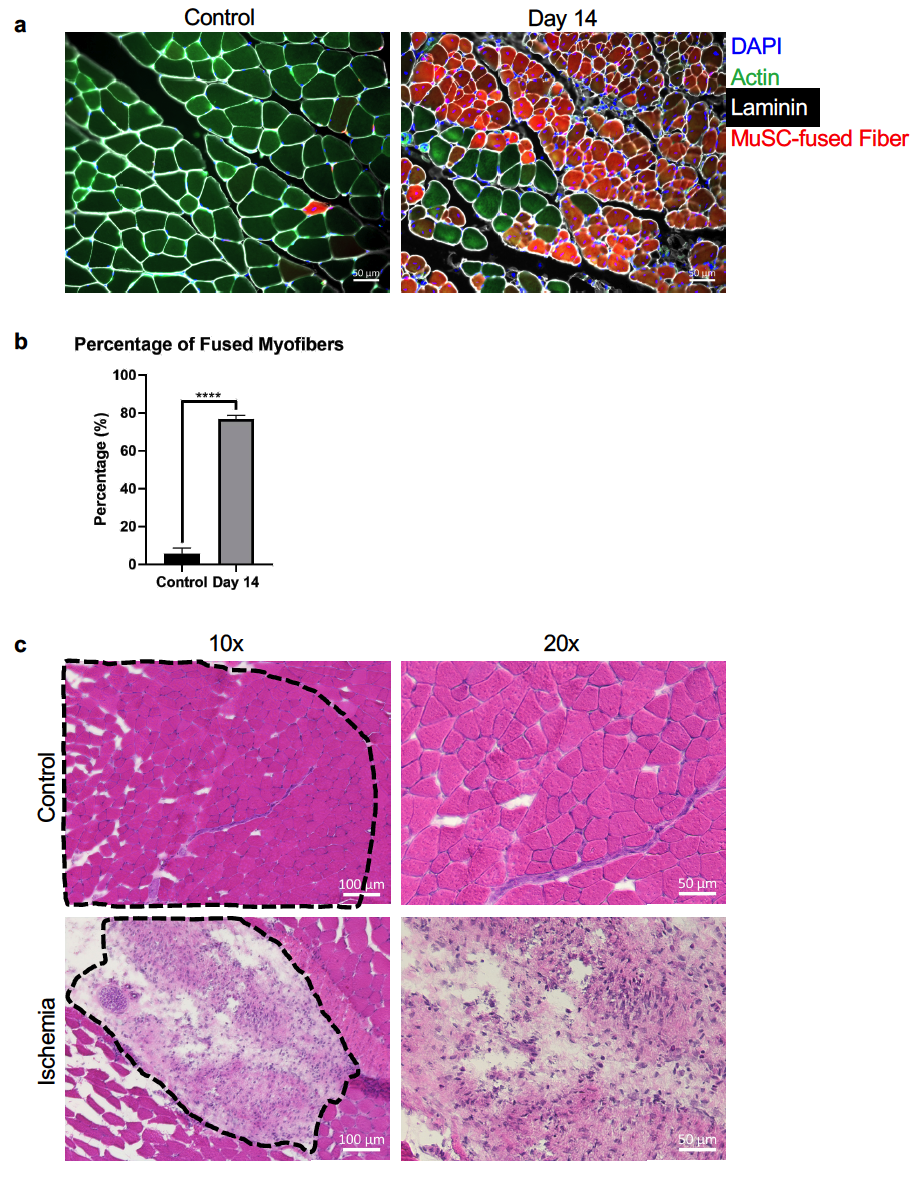
**

**Supplementary Figure S3. Muscle stem cell fusion following critical limb ischemia and myofiber damage in soleus of muscle stem cell depleted transgenic mice.** (a) Representative images of immunostained gastrocnemius muscle in *Pax7-tdTomato* transgenic mice 14 days following ischemic injury. Nuclei pseudo-colored in blue, actin in green, laminin in white, and MuSC-fused myofibers in red. (b) Quantification of the number of MuSC-fused myofibers 14 days following ischemic injury. n=3, *****p*<0.0001 compared to control. (c) Representative images of hematoxylin and eosin staining of soleus of *Pax7-DTA* transgenic mice, in which muscle stem cells are depleted. Soleus muscle is demarcated by the dashed outline and shows extensive myofiber damage following ischemic injury without muscle stem cells.

**
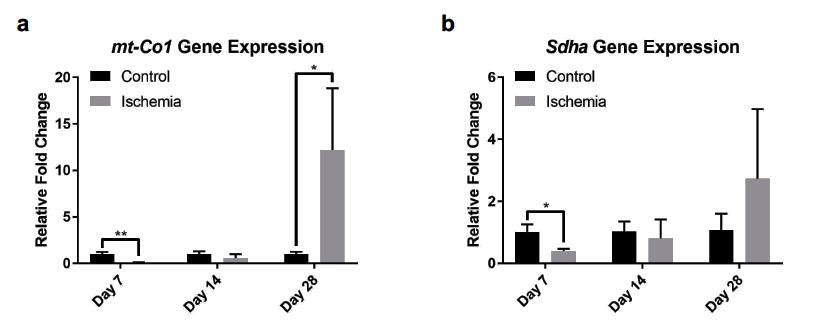
**

**Supplementary Figure S4. Mitochondrial Gene Expression.** (a) Relative gene expression of mitochondrial-encoded *mt-Co1* with quantitative PCR analysis at days 7, 14, and 28 following HLI. (b) Relative gene expression of nuclear-encoded *Sdha* with quantitative PCR analysis at days 7, 14, and 28 following HLI. n=3, **p*<0.05, ***p*<0.01 compared to control for all figures.

**
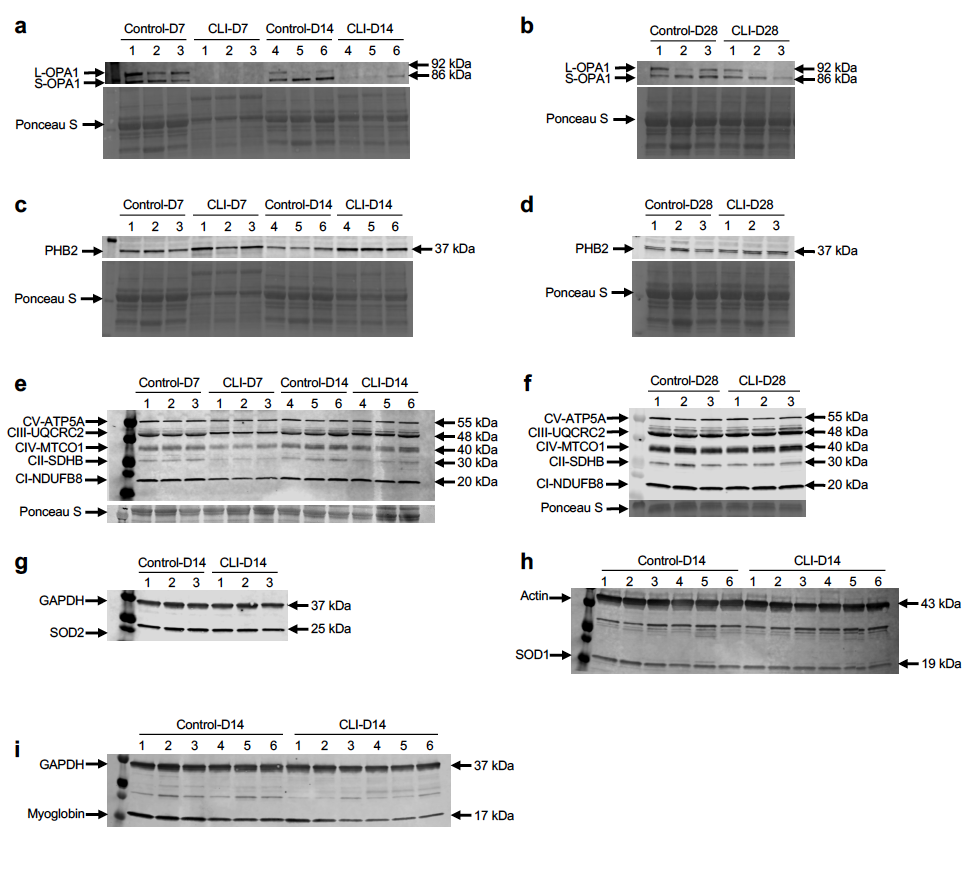
**

**Supplementary Figure S5. Western blot bands.** (a) Western blot analyses 7 days and 14 days following CLI for long (L-) and short (S-) isoforms of OPA1 and Ponceau S as loading control (n=3). (b) Western blot analyses 28 days following CLI for long (L-) and short (S-) isoforms of OPA1 and Ponceau S as loading control (n=3). (c) Western blot analyses 7 days and 14 days following CLI for PHB2 and Ponceau S as loading control (n=3). (d) Western blot analyses 28 days following CLI for PHB2 and Ponceau S as loading control (n=3). (e) Western blot analyses 7 days and 14 days following CLI for mitochondrial ETC complex I (NDUFB8-subunit), complex II (SDHB-subunit), complex III (UQCRC2-subunit), complex IV (MTCO1-subunit), complex V (ATP5A-subunit), and Ponceau S for loading control (n=3). (f) Western blot analyses 28 days following CLI for mitochondrial ETC complexes and Ponceau S for loading control (n=3). (g) Western blot analyses 14 days following CLI for SOD2 and GAPDH as loading control (n=3). (h) Western blot analyses 14 days following CLI for SOD1 and actin as loading control (n=6). (i) Western blot analyses 14 days following CLI for myoglobin and GAPDH as loading control (n=6). 3 or 6 representative biological samples for each blotted protein are shown and each protein blot was conducted on the same gel and membrane.

**
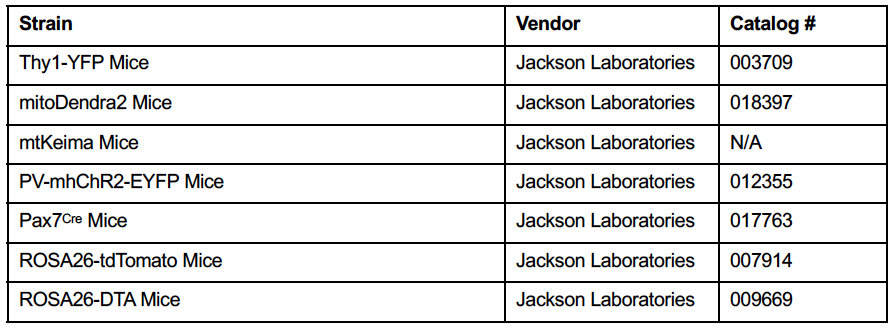
**

**Supplementary Table S1. List of transgenic mouse strains used in this study.**

**
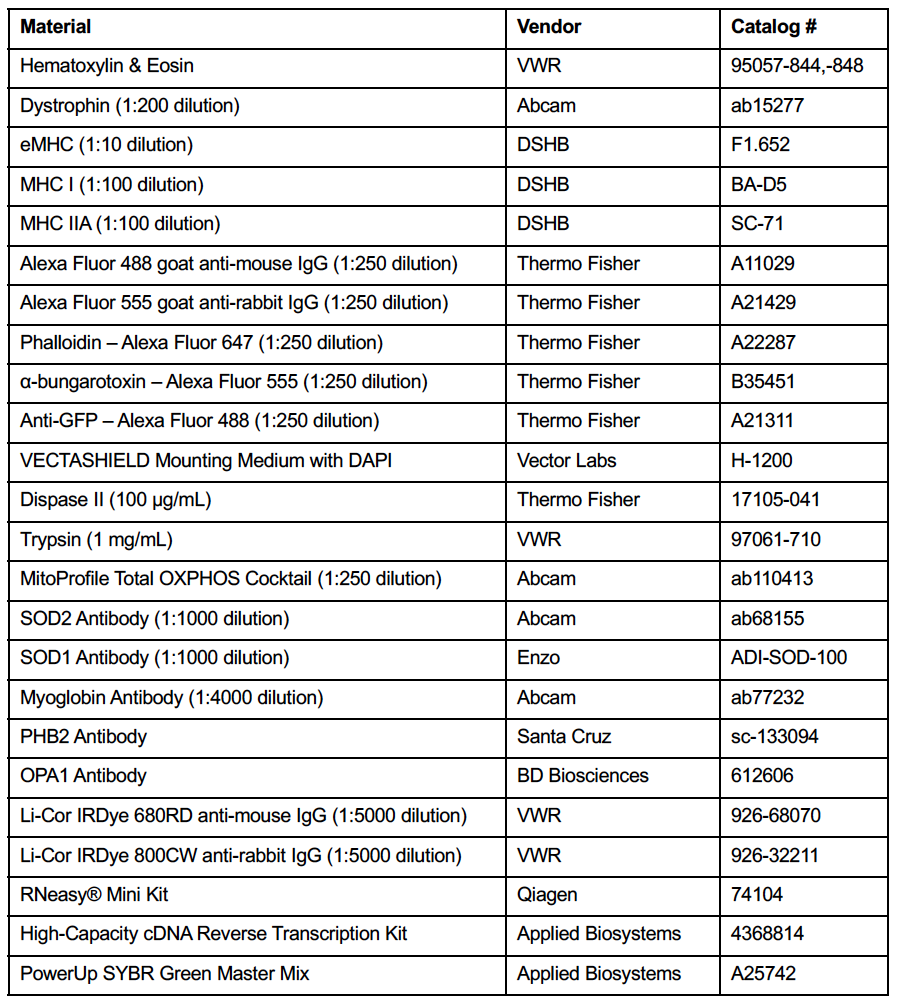
**

**Supplementary Table S2. List of materials, dilution factors, vendors, and catalog numbers used in this study.**

**
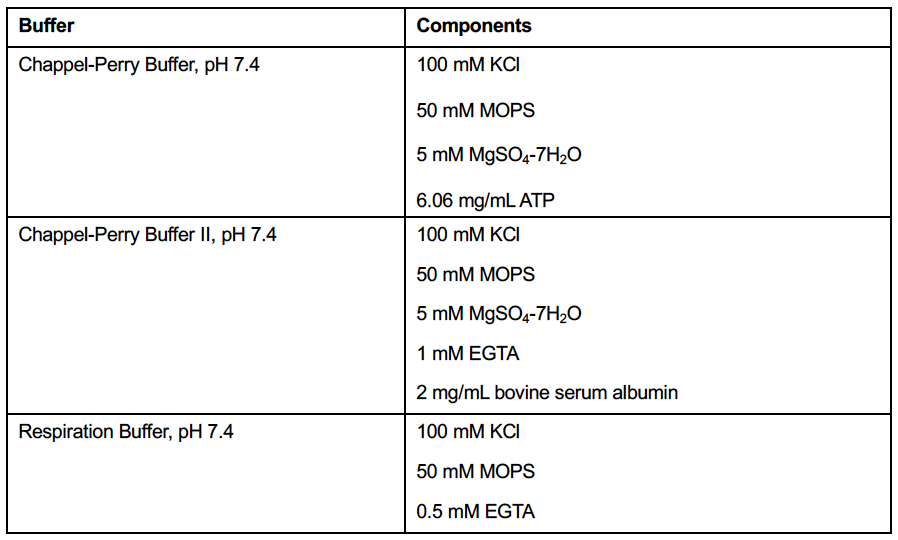
**

**Supplementary Table S3. List of buffers used for mitochondrial isolation and their respective components.**

**
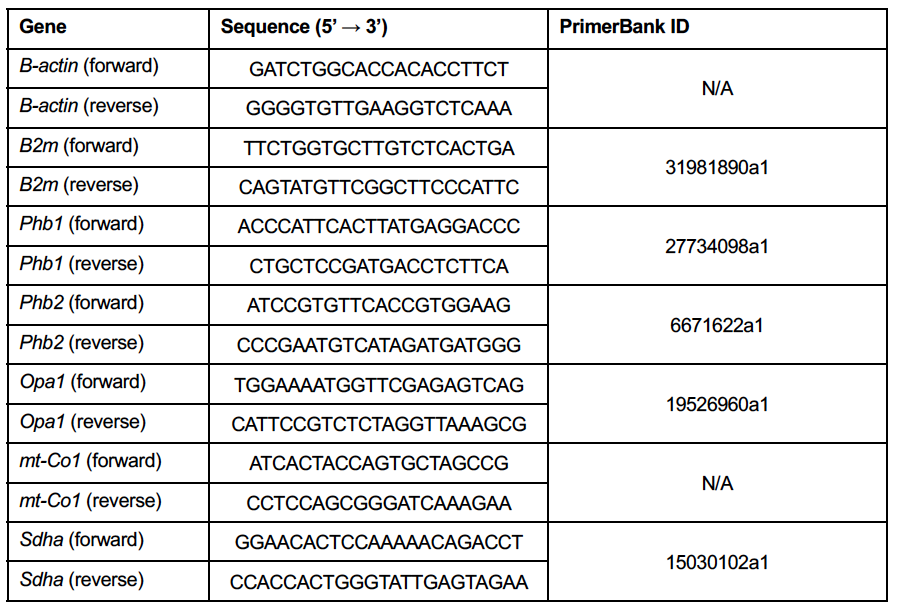
**

**Supplementary Table S4. List of gene sequences and PrimerBank ID’s used for each primer for quantitative polymerase chain reaction (qPCR).**
